# The hanging‐heart chip: A portable microfluidic device for high‐throughput generation of contractile embryonic stem cell‐derived cardiac spheroids

**DOI:** 10.1002/btm2.10726

**Published:** 2024-10-08

**Authors:** Pei‐Tzu Lai, Cheng‐Kun He, Chi‐Han Li, Jefunnie Matahum, Chia‐Yu Tang, Chia‐Hsien Hsu

**Affiliations:** ^1^ Institute of Biomedical Engineering and Nanomedicine National Health Research Institutes Miaoli County Taiwan, ROC; ^2^ Institute of Nanoengineering and Microsystems National Tsing Hua University Hsinchu Taiwan; ^3^ Ph.D. Program in Tissue Engineering and Regenerative Medicine National Chung Hsing University Taichung Taiwan

**Keywords:** cardiac spheroids, embryonic stem cell, hanging‐heart chip

## Abstract

Stem cell‐derived cardiac spheroids are promising models for cardiac research and drug testing. However, generating contracting cardiac spheroids remains challenging because of the laborious experimental procedure. Here, we present a microfluidic hanging‐heart chip (HH‐chip) that uses a microchannel and flow‐driven system to facilitate cell loading and culture medium replacement operations to reduce the laborious manual handling involved in the generation of a large quantity of cardiac spheroids. The effectiveness of the HH‐chip was demonstrated by simultaneously forming 50 mouse embryonic stem cell‐derived embryonic bodies, which sequentially differentiated into 90% beating cardiac spheroids within 15 days of culture on the chip. A comparison of our HH‐chip method with traditional hanging‐drop and low‐attachment plate methods revealed that the HH‐chip could generate higher contracting proportions of cardiac spheroids with higher expression of cardiac markers. Additionally, we verified that the contraction frequencies of the cardiac spheroids generated from the HH‐chip were sensitive to cardiotoxic drugs. Overall, our results suggest that the microfluidic hanging drop chip‐based approach is a high‐throughput and highly efficient method for generating contracting mouse embryonic stem cell‐derived cardiac spheroids for cardiac toxicity and drug testing applications.

**Translational Impact Statement**

In this study, the microfluidic hanging drop chip is a reliable 3D in vitro model for generating beating cardiac spheroids with high throughput and efficiency, ideal for further cardiac drug testing. This developed chip system serves as a potential model to simulate physiological in vivo conditions to study cardiac biology and disease mechanisms while reducing time and dependence on animal testing.

## 1 INTRODUCTION

Cardiac spheroids, three‐dimensional (3D) multicellular‐aggregate in vitro cell culture models, offer a more accurate representation of the cellular and extracellular heart microenvironments compared to conventional two‐dimensional (2D) cardiomyocyte cultures. They exhibit improved organotypic functionality, enhanced physiological response to stimuli, and refined responses.^1^, ^2^, ^3^ Owing to their ability to self‐renew and differentiate into many cell types, stem cells, including embryonic stem (ES) cells, have emerged as a promising source to generate multicellular cardiac spheroids and complex functional tissues.^4^, ^5^, ^6^ Stem cell‐derived cardiac spheroids have been applied in cardiotoxicity testing, cardiovascular and heart disease research, and drug development.^7^, ^8^, ^9^, ^10^ Using generated 3D artificial tissue, Takeda et al. predicted doxorubicin sensitivity and summarized the physiological properties of the cardiac‐related potassium channel‐blocking profile, in vitro.^9^ Richards et al. engineered an iPSC‐derived cardiac organoid model that resembled the vascular structure found within the developing myocardium and stimulated myocardial infarction and cardiotoxicity.^10^

The generation of 3D cardiac spheroids relies on cellular aggregation and self‐assembly in a scaffold‐free cell culture framework, where cell‐to‐device‐surface attachment is prevented.^11^, ^12^, ^13^, ^14^ Although this approach yields a high number of spheroids, they are not uniform in size and shape. To generate uniform cardiac spheroids, the hanging drop plate,^15^, ^16^, ^17^, ^18^ U‐shaped low‐attachment well plate,^19^, ^20^, ^21^, ^22^, ^23^, ^24^, ^25^, ^26^ and agarose mold^10^, ^27^, ^28^ methods have been adopted. Spheroids produced by the traditional hanging‐drop method are transferred to a low‐attachment plate to overcome the challenges of medium exchange within droplets. However, medium exchange in this approach is laborious, and extra care must be taken to prevent accidental spheroid damage or loss during medium dispensing and aspiration.

Microfluidic technology reduces the laborious manual handling of 3D cardiac spheroid cultures through the use of a microchannel and flow‐driven system, which facilitates cell loading and culture medium exchange operations. Wu et al. reported the development of a microfluidic chip‐based device that contains a microchannel with an array of opening wells, enabling high‐throughput culture of hanging droplet EB by hydrostatic pressure‐driven flow.^29^ The use of this microfluidic system facilitates the simultaneous formation of a large number of hanging droplets without the need to pipette the droplets individually. Additionally, Rismani et al. presented an integrated microfluidic hanging‐drop device containing an open‐hole row for iPSC‐derived cardiac spheroid culture, and a micropump with real‐time feedback flow control for medium circulation.^30^ Their study demonstrated the utility of microfluidic chips for the generation of beating cardiac spheroids in long‐term culture. Nevertheless, these microfluidic systems require connection to external pumps for medium exchange and drug testing, rendering scale‐up and setup challenging.

In this study, we demonstrate a novel 3D cardiac spheroid formation approach based on a portable microfluidic chip device and manual pipetting. This approach does not require an external pump, and the device has a small footprint, allowing for easy manual operation and enabling long‐term culture of cardiac spheroids in a stand‐alone device without a flow control system. The specific features of the HH‐chip compared to other cardiac spheroid generation methods were presented in Table 1.

**TABLE 1:** Comparison of cardiac spheroid generation methods.

Method	Cell type	Equipment	Beating	Beating ratio (%)	High‐throughput	Laborious handling	Spheroid uniformity	References
Rotating suspension cultures	ES‐CM	Rotating orbital shaker	Yes	Not claimed	Yes	No	No	[11]
	CM, ES‐EC, FB	Rotating orbital shaker	Yes	Not claimed	Yes	No	No	[12]
	iPS‐CM	Spinner flask	Yes	Not claimed	Yes	No	No	[13]
	iPS‐CM	Stirred‐tank bioreactor	Yes	Not claimed	Yes	No	No	[14]
Hanging‐drop	iPSC‐CM, EC, FB	Hanging‐drop plates, perfecta	Yes	Not claimed	Yes	Yes	Yes	[15]
	iPSC‐CM, FB	Hanging‐drop system, GravityPlus	Yes rate: 90 bpm	Not claimed	Yes	Yes	Yes	[16]
	CM, EC, FB	Hanging‐drop devices, elplasia	Yes	Not claimed	Yes	Yes	Yes	[17]
	ES	Hanging‐drop on the lid of dish	Yes	97%	Yes	Yes	Yes	[18]
Low‐adhesive agarose microwells	iPS‐CM, EC, FB	Agarose Molds with Hemispheric Microwells	Yes	Not claimed	Yes	Yes	Yes	[27]
	iPS‐CM, EC, FB	Agarose molds with hemispheric microwells	Yes	Not claimed	Yes	Yes	Yes	[10]
	CM, EC, or MSC	Agarose‐coated multiwells	Yes	40%	Yes	Yes	Yes	[28]
Ultra‐low multiwells plate	CM, EC, FB	Ultra‐low 96‐well plates	Yes rate: 12 bpm	Not claimed	Yes	Yes	Yes	[19]
	CM	Ultra‐low 96‐well plates	Yes rate: 54 bpm	Not claimed	Yes	Yes	Yes	[20]
	CM, EC, FB	Ultra‐low 384‐well plates	Yes rate: 60 bpm	Not claimed	Yes	Yes	Yes	[21]
	iPS‐CM, FB	Ultra‐low 96‐well plates	Yes rate: 30 bpm	Not claimed	Yes	Yes	Yes	[22]
	iPS‐CM	Microwell Plates, AggreWell	Yes	Not claimed	Yes	Yes	Yes	[23]
	CM, EC, FB	Ultra‐low 384‐well plates	Yes	Not claimed	Yes	Yes	Yes	[24]
	iPS‐CM, EC, SMC, FB	Ultra‐low 96‐well plates	Yes	Not claimed	Yes	Yes	Yes	[25]
	ES	Ultra‐low 6‐well plates	Yes	10%	Yes	Yes	Yes	[26]
Microfluidic‐controlled hanging‐drop	iPS	Pump‐integrated hanging‐drop chip	Yes rate: 60–90 bpm	Not claimed	No	No (need a pump)	Yes	[30]
Current study	ES	Portable HH‐chip	Yes rate: 60 bpm	90%	Yes	No	Yes	Pei‐Tzu Lai et al.

Abbreviations: CM, cardiomyocyte; ES, embryonic stem cell; EC, endothelial cell; ES‐CM, embryonic stem cell‐derived cardiomyocyte; ES‐EC, embryonic stem cell‐derived endothelial cell, FB, fibroblast; iPS‐CM, induced pluripotent stem cell‐derived cardiomyocyte, iPS, induced pluripotent stem cell, MSC, mesenchymal stem cell, SMC, smooth muscle cell.

## 2 RESULTS AND DISCUSSION

### 2.1 Device design

**FIGURE 1:**
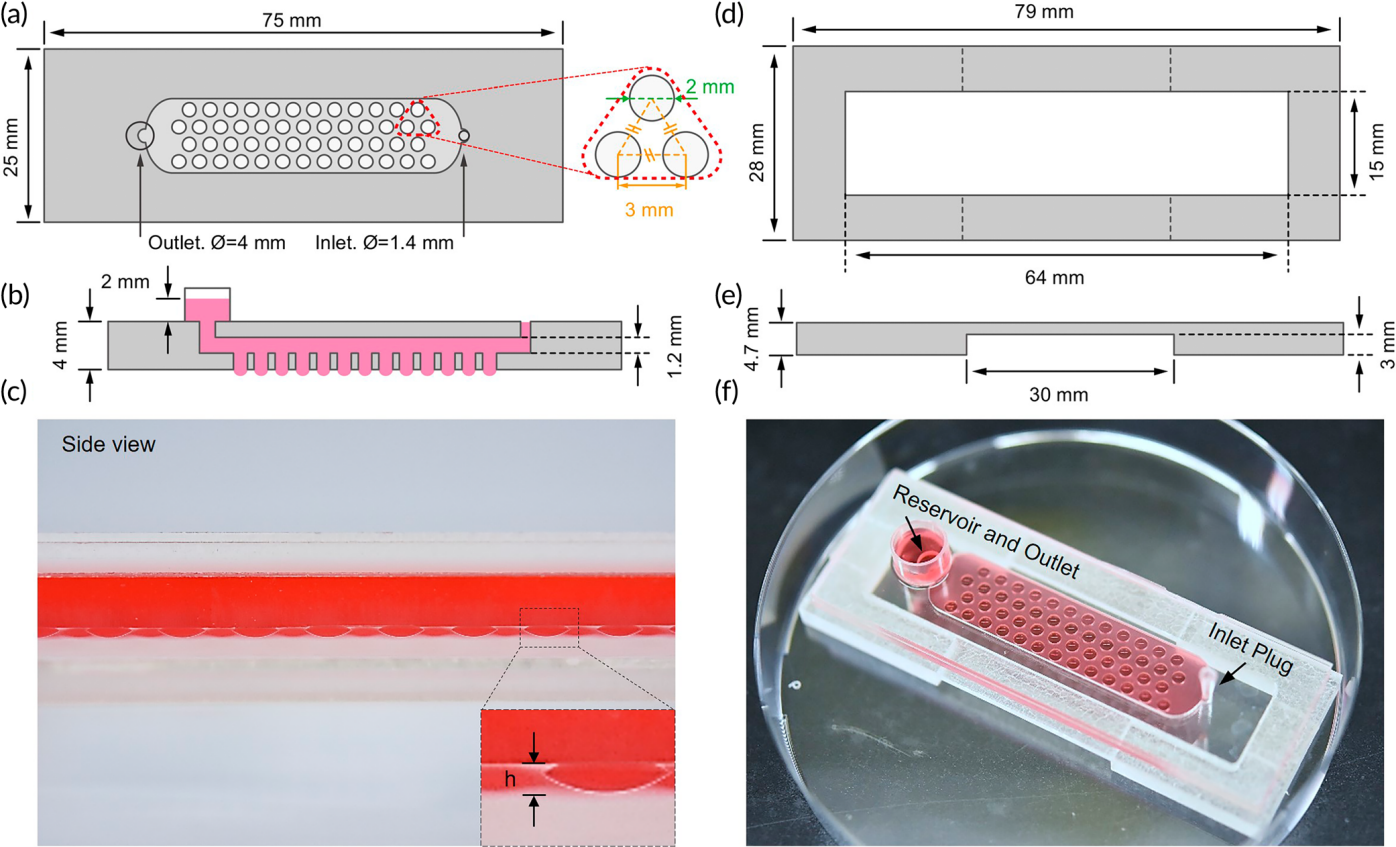
Microfluidic hanging‐heart chip (HH‐chip) design. (a) Top and (b) side views of the channel structure. (c) Photograph showing droplets held by hydraulic pressure. (d) Top and (e) side views of the rack structure used with the microfluidic device. (f) Photograph of the device filled with color dye, placed within a 10 cm dish.

In this study, a portable microfluidic chip was developed for the high‐throughput generation of cardiac spheroids. The designed chip includes a reservoir and an inlet hole on the chip, which allows the use of hydraulic pressure to maintain the hanging‐drop system. The pressure is easily controlled using a pipette and does not require any tubing or a pump. The chip consists of a channel containing an array of 50 through holes at its base for droplet generation, with two holes at the two ends of the channel serving as the inlet and outlet for medium injection, and a rack for the chip to sit on (Figure 1). The chip footprint is 75 × 25 mm and the through‐hole diameter is 2 mm. Based on previous data from our laboratory, an opening of 1.75 mm was found to be suitable for maintaining a stable embryoid body spheroid (~500 μm) in the hanging drop chip for 10 days of culture.^31^ For the necessary 15 days of culture in this study, the opening diameter was adjusted to 2 mm to prevent the spheroid (~800 μm) from touching the sidewall of the hole while maintaining stability of the hanging drop (i.e., without bursting) during device operation (Figure 1a, b). The inlet has a diameter of 1.4 mm to accommodate the tips of 1000 and 200 μL commercial pipettes. To prevent hydraulic changes during device movement, the inlet can be sealed with a homemade polyethylene plug. The outlet hole was 4 mm in diameter with a 10‐mm‐diameter reservoir on top of it. The outlet reservoir was used to hold the medium, exert hydrostatic pressure, and generate protruding hanging droplets from the through‐holes (Figure 1c). The channel and culture well have heights of 1.2 and 0.8 mm, respectively. A hollowed rack (79(*L*) × 28(*W*) × 4.7(*H*) mm) was designed to support chips, preventing droplets from coming into contact with the liquid that is added to the 10 cm dish (Figure 1d–f). However, the rack height exceeded the working distance of the observation, hindering high‐magnification photo capture on the microscope. Therefore, the rack height (4.7 mm) allowed the spheroids to be imaged with objectives of up to 10x magnification.

### 2.2 Device setup and operation

**FIGURE 2:**
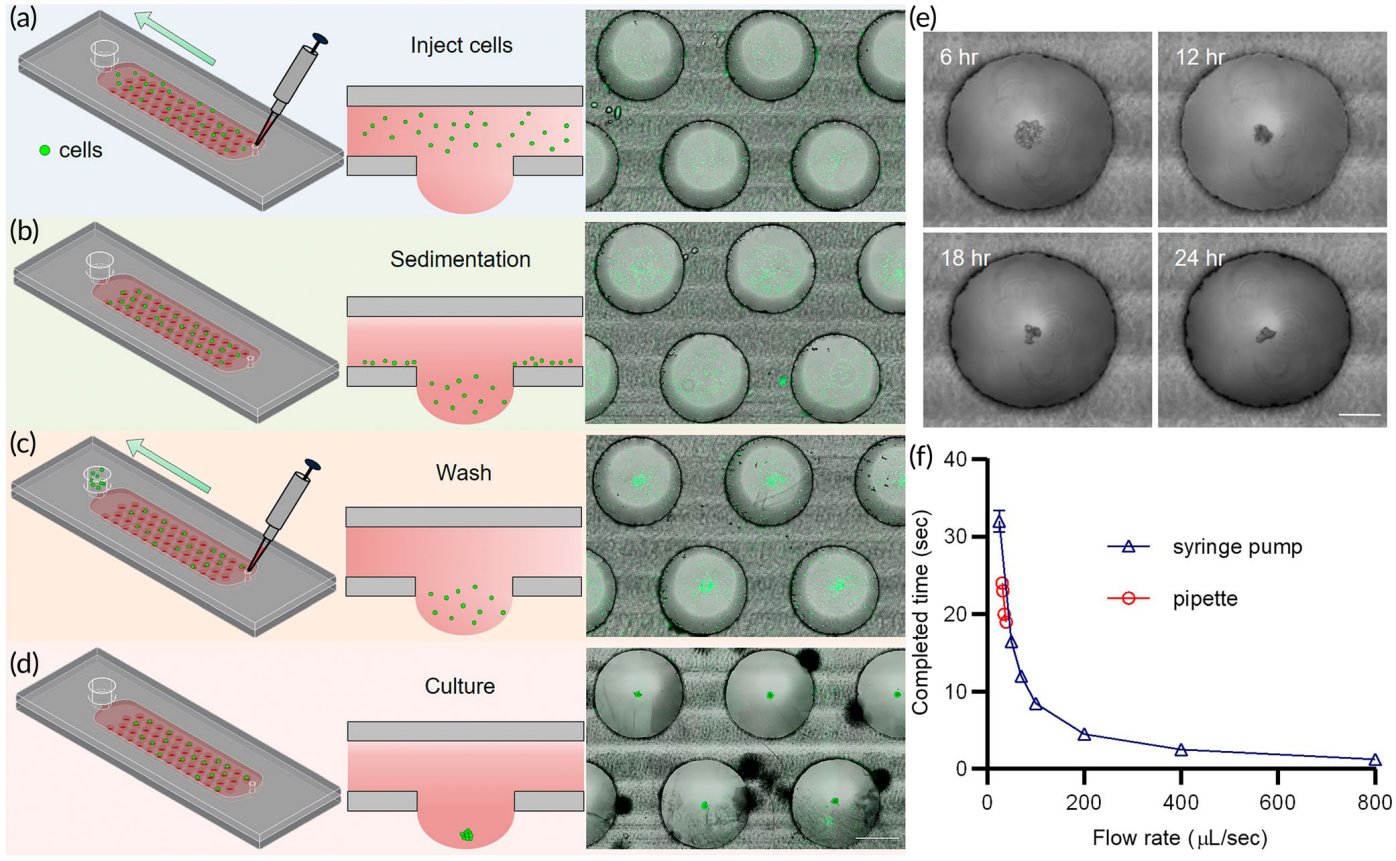
Operation procedure for the hanging‐heart chip. (a) Diagram of ES cell suspension loading into the channel at 3 × 10^4^ cells/mL. (b) Diagram of cell sedimentation at the bottom of well. (c) Diagram showing the washing of cells on the channel structure. Scale bar = 1000 μm. (d) Diagram of the formation of EBs after 24 h incubation. Scale bar = 1000 μm. (e) Cells aggregate on microfluidic chip after 24 h. Scale bar = 500 μm. (f) Graph depicting the relationship between flow rate and the time required to inject the culture medium into the chip. Data are presented as the mean ± SD, *n* = 3.

To start the cell culture experiment, the entire rack setup was placed inside a 10 cm petri dish. Subsequently, the microfluidic chip was positioned on top of the rack. PBS (10 mL) was added to the dish to prevent medium evaporation and maintain the moisture content. The cell suspension was added to the microchannel (Figure 2a) via the inlet hole using a manual pipette. After loading, the device was maintained for approximately 20 min to allow the cells to settle into the droplets (Figure 2b). To remove the excess cells, the channel was washed (six times) by aspirating 150 μL of medium from the reservoir and adding an equal volume of fresh medium into the channel inlet (Figure 2c). Culture medium was filled in the reservoir to a height of 2 mm to maintain the hydrostatic pressure required for the droplets to form EBs (Figure 2d). The culture wells were filled with culture medium, providing 28 μL of medium for each growing EB. Adjusting the hydrostatic pressure imposed by the liquid height within the reservoir is very important for generating and maintaining the stable spheroid on the chip over 15 days of culture. An increase in the liquid height within the reservoir leads to a rise in hydrostatic pressure, which in turn increases both the droplet height and its curvature. The enhanced curvature of a higher droplet allows cells to slide to the bottom of the droplet, promoting the formation of a single, stable hanging droplet. Conversely, the formation of two or three EB spheroids was observed within a single droplet, when the hydrostatic pressure was too low. In addition, the increased height of the droplet allows the weight of the liquid to overcome the surface tension, causing the droplet to burst.^29^ In this study, the height of the culture medium in the reservoir was adjusted to 2 mm to collect aggregated cells in the bottom center of the droplets, thereby enabling the preferential generation of only one spheroid in each droplet in this study (Figure 1b, c).

The operation steps of the microfluidic device, including cell loading and channel washing, are shown in Supplementary Movies 1 and 2. During the initial 24 h of incubation, ES cells underwent gravitational migration, to the bottom of the droplet and then formed a cell sphere (Figure 2e). Supplementary Movie 3 shows the sedimentation and aggregation of cells within the droplets from 0 to 48 h. Subsequently, the culture medium was replaced every 2 or 3 days, as described for the channel wash step. After culturing, the cardiac spheroids were harvested by lowering the HH‐chip into the PBS buffer in the dish, allowing the cardiac spheroids to fall into the buffer. The cells were subsequently collected for downstream analysis.

During the hanging drop chip operation, loading liquid at a high flow rate increases the risk of droplet bursting. High‐flow‐rate liquid loading results in increased pressure, exceeding the surface tension forces holding the droplet together, thereby causing the droplet to burst or detach from the chip. It is important to control the liquid loading and pressure to mitigate the risk of bursting droplets during hang‐drop chip operation. Using a syringe pump, the maximum tolerated flow rate (800 μL/s) was determined (Figure 2f). The manual pipette loading flow rate was then calculated by analyzing the video clips captured during the pipette operation experiments to ensure that the pressure in the HH chip was not exceeded during the liquid loading procedure. The manual pipette loading flow rate ranged from 31 to 35 μL/s, and the loading took less than 25 s. The results indicated that the flow rate created by the manual pipette was within the tolerance range. In addition, the results demonstrate that our portable device allows cells to be injected directly from the cell suspension into the HH chip via a pipette. Moreover, a simple manual operation helps avoid excessive flow rates, which could lead to droplet bursting during cell injection or when the culture medium is being replaced.

### 2.3 Generation of cardiac spheroids in the hanging‐heart chip

**FIGURE 3:**
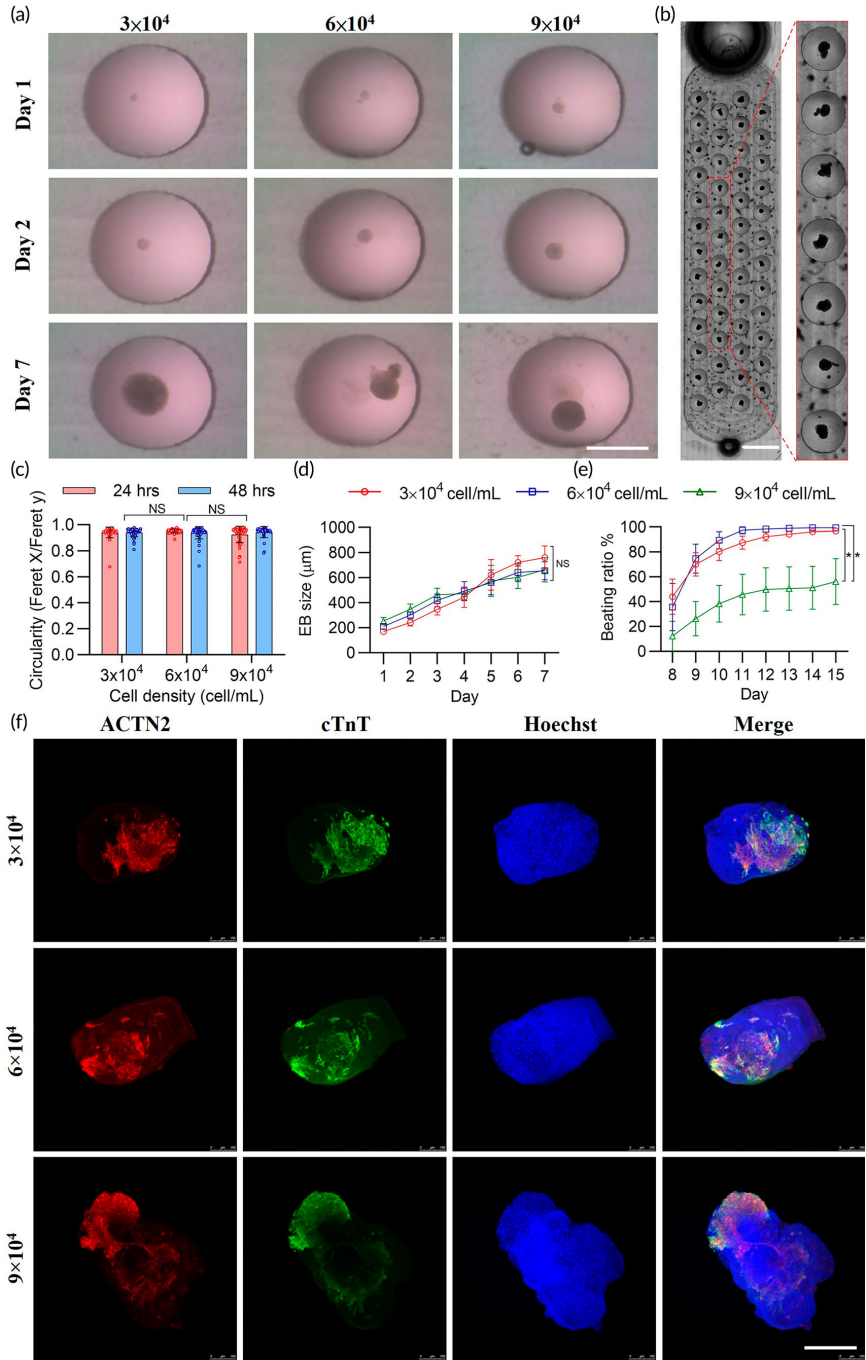
Cardiac spheroids generated on the heart‐hanging chip from mouse embryonic bodies using different cell densities. (a) Microscopy images showing EB morphology on ES culture on days 1, 2, and 7. Scale bar = 1000 μm. (b) Photograph of cell culture (initial cell load density = 3 × 10^4^ cells/mL) 15 days post‐incubation on the chip. Scale bar = 4000 μm. (c) Graph illustrating the circularity of EBs at 24 and 48 h post‐seeding (*n* = 60, three independent experiments). (d) Graph illustrating the size of EBs 7 days post‐culture (*n* = 60, three independent experiments). (e) Graph illustrating the beating ratio from days 8–15 (*n* = 6, three independent experiments). (f) Confocal images of EBs. Alpha actinin 2‐positive and cardiac troponin T‐positive cells are stained red and green, respectively, and nuclei are stained with Hoechst (blue) (*n* = 6, three independent experiments). Scale bar = 200 μm. CTN2, alpha‐actinin 2; cTnT, cardiac troponin T. The asterisks indicate statistical significance, *p <0.05; **p <0.01; ***p <0.001; NS, nonsignificant; error bars show standard deviation.

The differentiation outcomes of EB into contracting cardiac spheroids are dependent on homogeneity in EB size and morphology. Homogeneous EBs allow for the formation of contracting cardiac spheroids through directed ES cell differentiation in a more synchronous manner.^32^ Mouse embryonic D3 stem cells were used to generate uniformly sized cardiac spheroids. The initial size of the cell spheres was controlled by the loading density of D3 cells, as shown in Supplementary Figure 1. We found that D3 cells formed uniformly sized EBs within 24 h on the HH‐chip. Initially, the EB aggregates were round and compact on days 1 and 2, and they continued to grow until a diameter of approximately 600 μm was achieved by day 7 (Figure 3a). Spheroid circularity, calculated as the ratio of the short to long axes of the sphere, was approximately 0.9 (Figure 3c). The diameters of the spherical EBs formed from initial cell loads of 3 × 10,^4^ 6 × 10,^4^ and 9 × 10^4^ cells/mL were ~ 180, 200, and 220 μm, respectively, on day 1. Following EB formation, the cells were continuously cultured for several days (Figure 3b, Supplementary Movie 4). The EB size increased gradually over time, varying with different loading densities (Figure 3d).

We found that the device supported the generation of homogeneous EBs and their subsequent differentiation into cardiomyocytes with rhythmic contractions (Supplementary Movies [Link], [Link], [Link]). The formed EBs were incubated for 15 days in a culture well to calculate the beating ratio and analyze the expression of specific cardiomyocyte markers (Supplementary Figure 2). The beating ratio represents the percentage of beating spheroids over a total of 50 spheroids. For EBs derived from the initial cell loads of 3 × 10^4^ and 6 × 10^4^ cells/mL, beating occurred on day 8, with a beating ratio of greater than 90% by day 15. For EBs derived from the initial cell load of 9 × 10^4^ cells/mL, the beat ratio was <50% on day 15 (Figure 3e). This indicates that 3 × 10^4^ and 6 × 10^4^ cells/mL are appropriate initial loading densities for the generation of cardiac spheroids. Analysis of the cardiac markers, alpha‐actinin 2 (ACTN2) and cardiac troponin T (cTnT), revealed that the spheroids formed using the HH‐chip (Figure 3f) expressed both markers; however, ACTN2 and cTnT expression levels were found to be the highest in spheroids formed from the initial load of 6 × 10^4^ cells/mL (Supplementary Figure 3). The percentage of cardiomyocytes in individual spheroids was quantified by the intensity of cTnT and divided by the intensity of total cells stained with Hoechst. The findings revealed that the spheroids in the 6 × 10^4^ cells/mL group contained 67.6% cardiomyocytes, which was higher than the 37.1% cardiomyocytes in 3 × 10^4^ cells/mL group and 42.3% in the 9 × 10^4^ cells/mL group (Supplementary Figure 4). Therefore, based on the beating ratio and protein marker expression levels, we suggest that an initial cell density of 6 × 10^4^ cells/mL is optimal for EB differentiation.

Our data demonstrates that the HH‐chip generates a uniform cardiac spheroid with optimal size and uniform circularity at the optimized cell density, and subsequently promotes further differentiation, into contracting cardiac spheroids with a higher beating ratio and cardiac marker expression.

### 2.4 Comparison of cardiac spheroids formed using the HH‐chip, the hanging‐drop method, and low‐attachment plates

**FIGURE 4:**
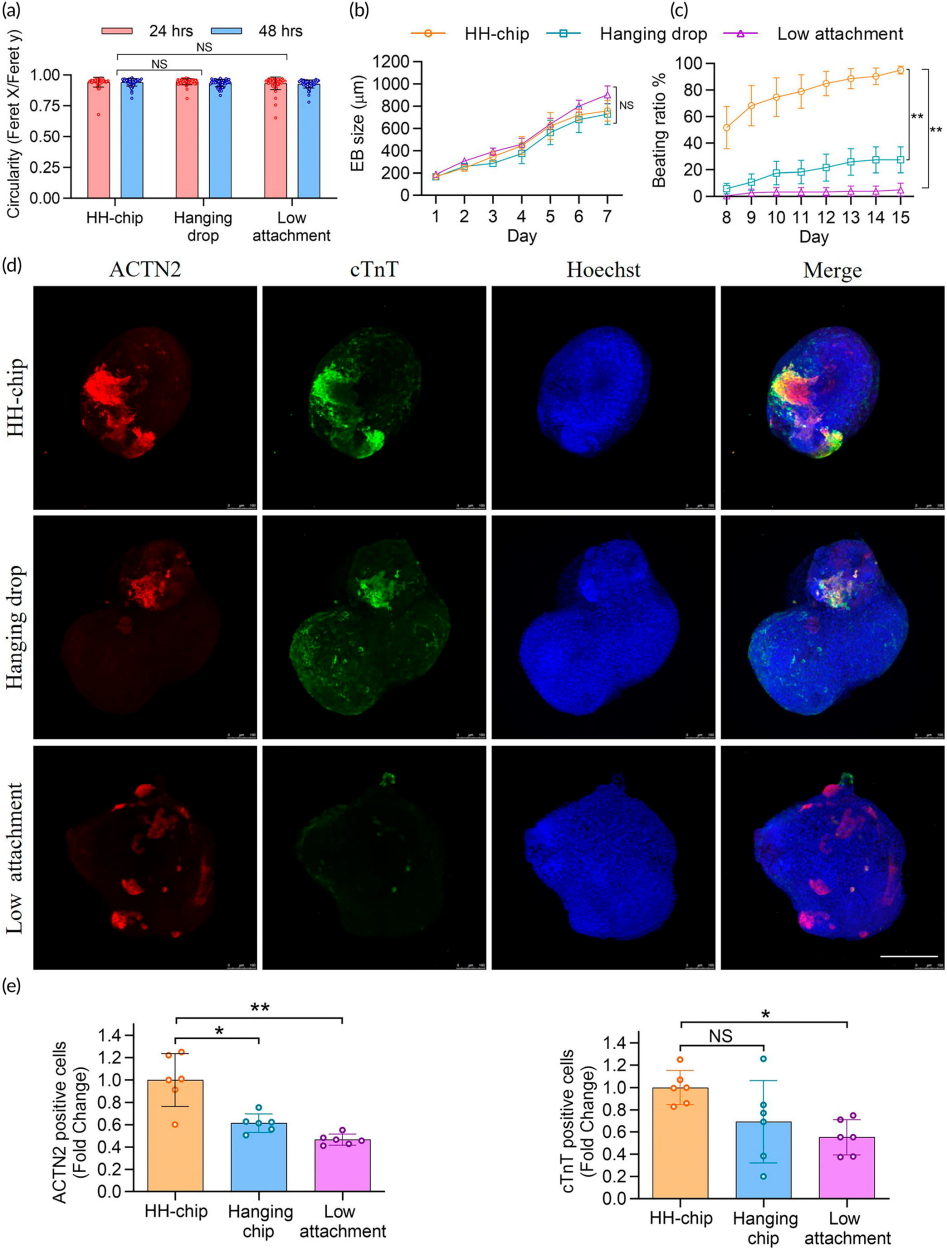
Comparison of embryonic bodies formed using the heart‐hanging chip, traditional hanging‐drop, and low‐attachment 96‐well plate methods. (a) Bar graphs showing EB circularity at 24 and 48 h (*n* = 60, three independent experiments). (b) Graphs showing the change in EB size over a 7‐day culture period (*n* = 60, three independent experiments). (c) Graphs showing the beating ratio from days 8–15 (*n* = 6, three independent experiments). (d) Confocal images of EBs showing protein expression (*n* = 6, three independent experiments). Scale bar = 200 μm. (e) Bar graphs illustrating confocal image quantification of protein expression (*n* = 6, three independent experiments). For normalization, the intensities of ACTN2 and cTnT were normalized to the total cell number stained with Hoechst. Subsequently, the data were presented as fold changes, with the value of HH‐chip set to one‐fold. Data from the other two methods are indicated relative to this value. ACTN2, alpha‐actinin 2; cTnT, cardiac troponin T. The asterisks indicate statistical significance, *p <0.05; **p <0.01; ***p <0.001; NS: nonsignificant; NError bars show standard deviation.

We compared three different cardiac spheroid culture methods: our developed method, the conventional hanging drop method, and the low‐attachment plate method. After 24 h of culturing, the size and circularity of the spheres were similar for each method (Figure 4a). We further analyzed the cystic structures and fluid‐filled cavities similar to blastocysts, which differentiate into early extraembryonic tissue as a morphologic characteristic at the onset of differentiation.^33^, ^34^ Both the EBs formed on the HH‐chip and the low‐attachment 96‐well plate presented as cystic structures on day 7 (Supplementary Figures 5 and 6), with diameters of 900 μm (Figure 4b). EBs formed using the traditional hanging‐drop method were < 700 μm on day 7 and did not exhibit a cystic structure. The absence of cystic structures may be attributed to the cell spheres that easily touched and adhered to the supporting surface during medium changes (Supplementary Figure 7).

The beating ratio differed among EBs formed by the different methods (Supplementary Movies 8 and 9). On day 15, the beating ratio of EBs formed by the traditional hanging drop and low‐attachment 96‐well plate methods was <40% (Figure 4c). A beating ratio of >90% was observed for EBs formed on HH chips. A comparison of our method with the other two methods revealed that all methods could generate uniform EBs, in terms of circularity; however, the beating ratio was significantly higher in EBs generated using our system. Beating is a functional characteristic of mature cardiac differentiation. A similar phenomenon of a low cardiac spheroid beating ratio has been previously observed^26^ with the use of the low‐attachment plate method. However, the cause of this phenomenon remains unclear. In the traditional hanging‐drop group, we speculate that the main reason for the lower beating ratio was poor differentiation at an early stage, as proven by the absence of cystic structures.

Additionally, confocal microscopy analysis of cardiac marker (ACTN2 and cTnT) expression (Figure 4d) revealed significantly higher levels of these proteins in EBs formed using the microfluidic method (Figure 4e). The percentage of cardiomyocytes in individual spheroids was also determined by comparing the intensity of cTnT to the intensity of total cells, stained with Hoechst. The results indicated that the HH‐chip method produced spheroids with 33.8% cardiomyocytes, which was higher than the 24.1% observed in the traditional hanging drop method and 19.3% in the low attachment method (Supplementary Figure 8). These results indicate that, in the HH‐chip, spheroid differentiation was improved. Our results indicated that improved differentiation is likely responsible for a higher beating ratio. The HH‐chip offers a more stable culture microenvironment, minimizing spheroid disruption during cardiomyocyte differentiation, thereby increasing further differentiation characteristics such as cystic structures, marker expression, and high beating ratio. However, the mechanism underlying this promotion requires further investigation.

### 2.5 Drug responses of cardiac spheroids

**FIGURE 5:**
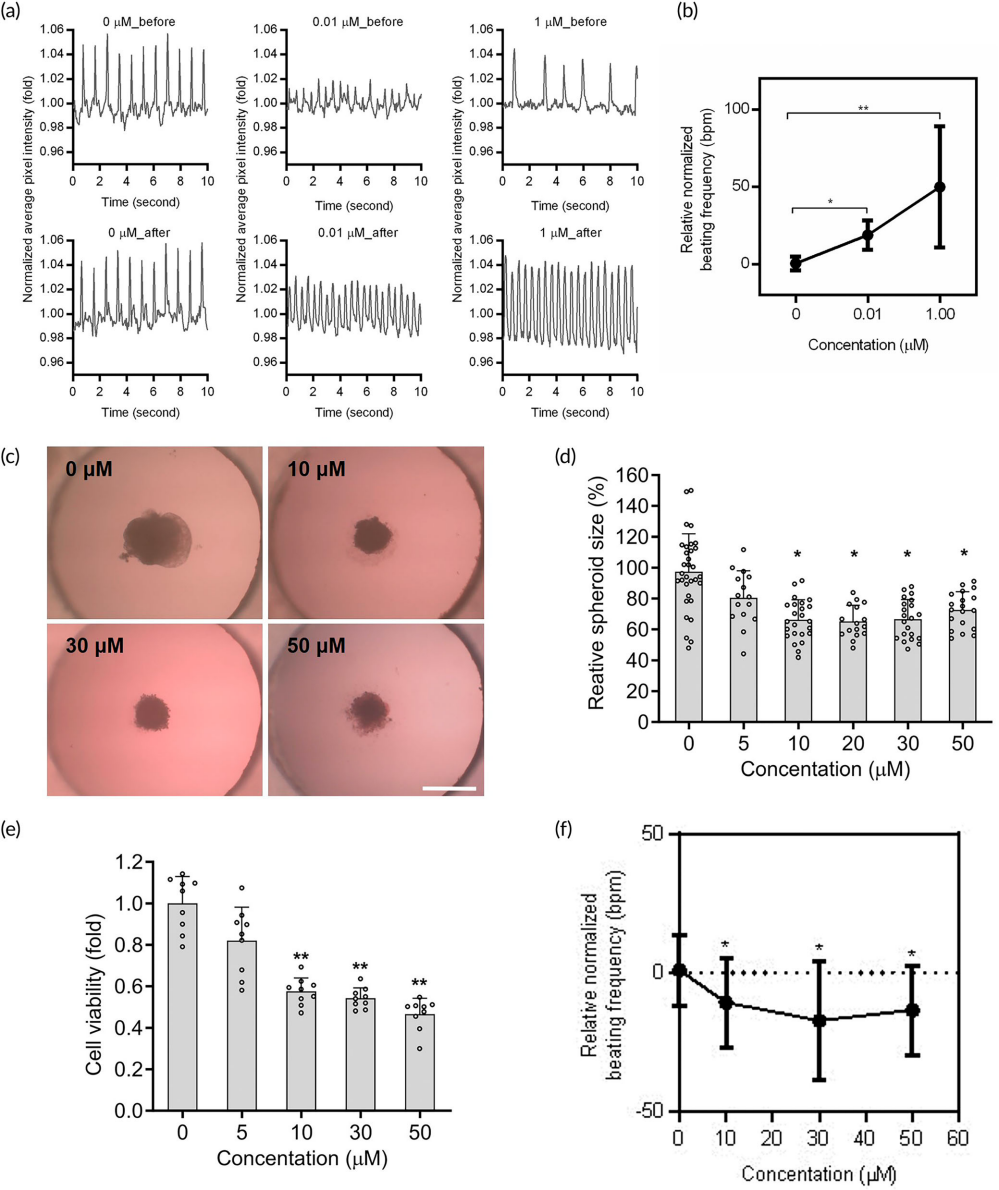
Effects of drug‐induced cardiotoxicity on embryonic bodies formed using our hanging‐heart chip. (a) Graphs showing the frequency of beating of EBs with different patterns, and the frequency of beating EBs treated with 0, 0.01, and 1 μM isoproterenol for 10 min. (b) Quantification of the beating frequency changes before and after isoproterenol treatment. Data are presented as the mean ± SD, *n* = 6, in three independent experiments. (c) Microscopy images show morphology of EBs following exposure to 10, 30, and 50 μM doxorubicin. Scale bar = 500 μm. (d) Quantification of the spheroid size with doxorubicin treatment. Data are presented as the mean ± SD, *n* >15, in three independent experiments. (e) Quantification of cell viability with doxorubicin treatment. Data are presented as the mean ± SD, *n* = 9, in three independent experiments. (f) Graph showing the normalized beating frequency after exposure to 0–50 μM doxorubicin (*n* = 15, three independent experiments).

A cardiotoxicity test was used to assess the potential of the chip‐generated cardiac spheroids as a promising in vitro model for cardiotoxicity drug response evaluation. Thus, the response of cardiac spheroids to isoproterenol and doxorubicin was demonstrated in the HH‐chip by recording a beating video of EBs and analyzing the frequency of beating.^35^ The analysis was presented with a beating graph both before and after isoproterenol treatment. The control group (0 μM) comprised spheroids cultured in the medium without isoproterenol. The drug testing data showed that the beating frequency of each EB varied considerably; however, treatment with isoproterenol resulted in a significant increase in beating frequency at doses exceeding 0.01 μM (Figure 5a). Quantification of these changes in beating frequency is detailed in Figure 5b, demonstrating that isoproterenol considerably enhanced the beating frequency at doses over 0.01 μM with no significant difference observed between the 0.01 μM and 1 μM groups. Additionally, videos illustrating the beating spheroid before and after isoproterenol treatment were also provided to visually demonstrate the ability of isoproterenol to increase beating frequency (Supplementary Movies [Link], [Link], [Link], [Link], [Link], [Link]).

Doxorubicin caused a decrease in the size of the EBs when exposed to dosages over 10 μM (Figure 5c, d). Additionally, the WST‐1 cell viability assay was employed to examine the cardiotoxic effects of doxorubicin (Figure 5e). The data indicated that doxorubicin exhibits a significant decrease in cell viability following treatment with 5 μM doxorubicin for 48 h. The WST‐1 assay data further indicated that the spheroids treated with doxorubicin had dysfunctional mitochondria, as evidenced by decreased activity of mitochondrial dehydrogenases. Moreover, the beating frequency of the spheroids was significantly reduced with a 10 μM doxorubicin treatment (Figure 5f). These findings collectively demonstrate that doxorubicin reduces cell viability, mitochondrial activity, spheroid size, and beating activity. These findings demonstrate that chip‐generated cardiac spheroids provide promising capabilities for cardiotoxicity drug studies.

In this study, a portable microfluidic device was designed to efficiently generate contractile cardiac spheroids for cardiotoxic drug testing. The in vitro model of the chip‐generated cardiac spheroids demonstrated promising capabilities for cardiac‐related studies, drug discovery, and safety pharmacology.

## 3 CONCLUSION

As a potential model, 3D culture systems of cardiomyocytes can be used to simulate physiological in vivo conditions, which is useful in the study of cardiac biology and disease; such systems reduce time consumption and dependence on animal testing. Our device allows for manual loading with a micropipette to generate droplets of the array from the channel of the chip using hydrostatic pressure, eliminating the need for tubing or pumps. It provides a simple method for the high‐throughput generation of cardiac spheroids and reduces laborious manipulation during medium replacement. Using D3 mouse ES cells, we demonstrated that the microfluidic hanging drop chip‐based approach can generate beating cardiac spheroids on‐chip with high throughput and efficiency. This platform is a promising technique for building reliable 3D in vitro models for drug development.

## 4 METHODS

### 4.1 ES cell culture and differentiation

Mouse embryonic D3 stem cells (provided by Professor Shaw Fang Yet, National Health Research Institutes) were maintained in an undifferentiated state by culturing them on mitotic, inactive mouse embryonic fibroblast feeder cells (BCRC Cat.M‐EF001). The cell culture medium was Dulbecco's Modified Eagle Medium (Gibco, CA, USA) supplemented with 15% fetal bovine serum (Hyclone, Australia), 2 mM glutamax (Gibco, CA, USA), 0.1 mM nonessential amino acids (Corning Inc., NY, USA), 0.1 mM 2‐mercaptoethanol (Gibco, CA, USA), and 2000 U/mL mouse murine leukemia inhibitory factor (LIF; Millipore, Burlington, USA). The differentiation medium was culture medium without LIF. For cardiomyocyte differentiation, EBs were formed in the D3 cell suspension and differentiated in a 3D culture system for 15 days. The medium was replaced with fresh culture medium every 2–3 days, and ES‐differentiated cardiomyocytes were identified by their hallmark rhythmic contractions.

### 4.2 Device fabrication and operation

The HH‐chip and device rack were made from polystyrene and poly (methyl methacrylate), respectively. We designed a channel structure using AutoCAD software (Education Edition, Mill Valley, USA), and the chips and rack were sliced using a computerized numerical control machine (Roland, MDX‐40, Irvine, CA, USA). The microfluidic chips consisted of two layers that were bonded together using double‐sided tape (3M™ Ultra‐clean Laminating Adhesive 502FL, Maplewood, USA), that was cut using a laser engraving machine (FLUX Delta, Taipei, Taiwan). D3 cells, cultured in differentiation medium, were counted and diluted to a density of 3 × 10,^4^ 6 × 10,^4^ and 9 × 10^4^ cell/mL. Next, the cells (800 μL) were loaded into a HH‐chip. After loading, cells were allowed to settle into the droplet for 20 min (Figure 2b). Then, the channel was washed by the removal and replacement of the reservoir medium (150 μL) (Figure 2c). This wash step was repeated six times. Next, to enable cellular aggregation into spheres, the cells were incubated for 24 h. The culture medium was replaced every 2–3 days; 100 μL of the reservoir medium was removed and an equal volume of fresh differentiation medium was added into the inlet. This step was repeated 10 times to ensure sufficient hydraulic pressure and prevent cell sphere disturbance and contact with the sidewall. Gentle changes in the medium did not affect EBs (Supplementary Movie 16).

### 4.3 3D culture system

To compare spheroid formation across HH‐chips, hanging drop, and low attachment methods, identical initial cell numbers were used in each method. Initially, the cell number in the HH‐chip well was directly calculated from an image captured under a microscope (Supplementary Figure 1). Subsequently, the same number of cells were seeded for the hanging drop and low attachment methods. For hanging‐drop culture, we seeded 600 ES cells into 20 μL of differentiation medium. The medium was replaced every 2 or 3 days; the medium was removed and replaced with fresh medium (without touching the sphere). For culture in the low‐attachment 96‐well plate (Ultra‐low, Corning Inc.), we seeded 600 ES cells into each well. The cells were cultured in 200 μL differentiation medium. The medium was replaced with a fresh medium every 2 or 3 days.

### 4.4 ELISA

The ELISA was performed using the commercial mouse cTnT (CUSABIO, Houston, TX, USA) and mouse ACTN2 (BlueGene, Shanghai, China) ELISA kits.

### 4.5 Immunostaining

For immunofluorescence staining, the differentiated spheres were fixed with 4% paraformaldehyde (Sigma‐Aldrich) for 30 min and washed three times with PBS containing 0.1% Tween 20. The spheres were then permeabilized for 30 min using 1% Triton‐X100 in PBS, washed three times, and blocked by incubation with 5% bovine serum albumin (in 0.5% Triton‐X100‐PBS overnight). After washing with PBS, cells were incubated with primary antibodies, including mouse anti‐cTnT (Abcam, Cambridge, UK) and rabbit anti‐ACTN2 (Proteintech, Chicago, USA), overnight at 4°C. Following incubation, the cells were washed three times using PBS containing 0.1% Tween 20. Secondary antibodies, including goat anti‐mouse fluorescein isothiocyanate (Abcam, Cambridge, UK) and Alexa Fluor 594 donkey anti‐rabbit (Invitrogen, Waltham, USA), and a DNA stain (Hoechst 33342, Sigma, MA, USA), were added to the cells and incubated for 5 h at room temperature. Following incubation, the cells were washed with PBS, and the spheroids were examined using a confocal microscope.

### 4.6 The percentage of cardiomyocyte quantification

The percentage of cardiomyocytes in individual spheroids was determined by comparing the intensity of ACTN2 or cTnT to the intensity of total cells, stained with Hoechst. Given that the expression of cTnT was higher than that of ACTN2 in the HH‐chip results, cTnT data was used to represent the percentage of cardiomyocytes in this study.

### 4.7 Drug testing

The 3D cardiac spheroids generated by microfluidic devices were stimulated by isoproterenol (Sigma‐I5627) and doxorubicin hydrochloride (Sigma‐44583) treatments. Spheroids differentiated from D3 cells, that had been seeded at 3 × 10^4^ cells/mL and cultured for 12 days, were induced by isoproterenol exposure (0–1 μM) for 10 min on the microfluidic device. Spheroid, differentiated from D3 cells and cultured for 10 days, were exposed to doxorubicin (0–50 μM) for 48 h to induce toxicity on the microfluidic device.

### 4.8 Image acquisition

Microfluidic device, traditional hanging‐drop, and low‐attachment 96‐well plate images were captured using a Nikon SMZ1500 stereoscopic zoom microscope. Large‐image and time‐lapse were performed with Nikon eclipse Ti2. The confocal images were captured using the Leica TCS SP5 inverted fluorescence microscope.

### 4.9 Image analysis

The diameter and circularity of spheroids were analyzed using NIH ImageJ and Nikon NIS software. The ATCN2 and cTnT positive areas, as proportions of the total area, were analyzed by evaluating the black‐to‐white ratio using NIH ImageJ software.^15^

### 4.10 Frequency of beating analysis

Videos of cardiac spheroids were recorded using a SAGE VISION SGHD‐3600 HDMI camera (SAGE VISION Co., Ltd., Taipei, Taiwan) at a frame rate of 25 frames/s. The region of interest with beating was selected using ImageJ software, and dynamic pixel changes were used to generate graphs illustrating cardiac beating (Supplementary Figure 9). Origin 9.1 software was used to calculate the beating peak time, which was more than 60% higher when compared with Minimax.^35^ The time‐to‐peak was exported to Microsoft Excel to calculate beats/min. The normalized average pixel intensity at each time point was normalized, as detailed in the following equation.

$$\text{Normalized average pixel intensity}\left(\text{fold}\right)=\frac{\text{Average intensity of each pixel in the}\;\mathrm{ROI}\;\text{of individual frame}}{\text{Average intensity of each pixel in the}\;\mathrm{ROI}\;\text{of total frames}}.$$

### 4.11 WST‐1 assay

The cell viability of spheroid with doxorubicin treatment was evaluated using the WST‐1 assay. After treatment for 48 h, the spheroids were collected to the 96 well plate and washed with phosphate buffer solution and then added to 100 μL of fresh medium and 10 μL of Cell Proliferation Reagent, WST‐1 (Roche Diagnostics, Germany) that would react for 4 h at 37°C. The medium was collected from the culture dish and analyzed using an ELISA reader (Molecular Devices, USA). The absorbance at 450 nm was recorded as the OD value in terms of cell viability.

### 4.12 Statistical analysis

Statistical and graphic analyses were performed using GraphPad Prism 6 software. All experiments were conducted at least in triplicate. One‐way analysis of variance and Tukey's multiple comparison tests were used to compare groups.

## AUTHOR CONTRIBUTIONS

**Pei‐Tzu Lai:** Conceptualization; data curation; formal analysis; investigation; methodology; project administration; resources; validation; visualization; writing – original draft; writing – review and editing. **Cheng‐Kun He:** Conceptualization; data curation; formal analysis; investigation; methodology; project administration; validation; visualization; writing – original draft; writing – review and editing. **Chi‐Han Li:** Conceptualization; data curation; formal analysis; investigation; methodology; project administration; validation; visualization; writing – original draft; writing – review and editing. **Jefunnie Matahum:** Data curation; formal analysis; investigation; methodology; project administration; validation; visualization. **Chia‐Yu Tang:** Data curation; formal analysis; investigation; methodology; project administration; validation; visualization. **Chia‐Hsien Hsu:** Conceptualization; supervision; writing – original draft; writing – review and editing.

## FUNDING INFORMATION

This work was supported by a grant from the Ministry of Science and Technology (MOST109‐2221‐E400‐003‐MY2), and National Health Research Institutes.

## CONFLICT OF INTEREST STATEMENT

The authors declare that they have no conflict of interest.

### PEER REVIEW

The peer review history for this article is available at https://www.webofscience.com/api/gateway/wos/peer-review/10.1002/btm2.10726.

## Supporting information


**Supplementary Figure 1.** Images of ES cells suspension in the microfluidic chip at (a) 3 × 10,^4^ (b) 6 × 10,^4^ and (c) 9 × 10^4^ cell/mL loading densities. Scale bar = 1000 μm.
**Supplementary Figure 2.** Photographs of ES spheroids cultured in the microfluidic chip for 7 days at (a) 3 × 10,^4^ (b) 6 × 10,^4^ and (c) 9 × 10^4^ cell/mL loading densities, and for 15 days at (d) 3 × 10,^4^ (e) 6 × 10,^4^ and (f) 9 × 10^4^ cell/mL loading densities. Scale bar = 4000 μm.
**Supplementary Figure 3.** (a) ACTN2 and (b) cTnT protein expression of cardiac spheroids cultured in the HH‐chip for 15 days. Data are presented as the mean ± SD, *n* >19, in three independent experiments.
**Supplementary Figure 4.** The percentage of cardiomyocytes in individual spheroids was quantified by the intensity of (a) ACTN2 and (b) cTnT divided by the intensity of total cells stained with Hoechst at different cell seeding densities. Data are presented as the mean ± SD, *n* = 6, in three independent experiments.
**Supplementary Figure 5.** Images of ES cells cultured by using HH‐Chip (left column), traditional hanging drop (middle column), and low‐attachment 96‐well plate (right column) methods. Scale bar = 1000 μm.
**Supplementary Figure 6.** Photographs of ES spheroids formed in the commercial low‐attachment 96‐well plate after 7 days of culture. Scale bar = 10 mm.
**Supplementary Figure 7.** Micrographs of mouse ES‐formed spheroids in traditional hanging drops for 7 days. The arrows indicate the spheroids that attached to the supporting surface due to manual pipetting during medium change. Scale bar = 1000 μm.
**Supplementary Figure 8.** The percentage of cardiomyocytes in individual spheroids was quantified by the intensity of (a) ACTN2 and (b) cTnT divided by the intensity of total cells stained with Hoechst in three spheroid culture methods. Data are presented as the mean ± SD, *n* = 6, in three independent experiments.
**Supplementary Figure 9.** (a) The beating frequency of a cardiac spheroid measured at the selecting region which is shown as the dashed circle in (b).

**Supplementary video 1** Chip operation.

**Supplementary video 2** Cell loading and sedimentation.

**Supplementary video 3** Sphere formation during 0–48 h.

**Supplementary video 4** EB culture on chip from day3 to day6 timelapse.

**Supplementary video 5** The beating of cardiac spheroid at 3‐10^4 loading density.

**Supplementary video 6** The beating of cardiac spheroid at 6‐10^4 loading density.

**Supplementary video 7** The beating of cardiac spheroid at 9‐10^4 loading density.

**Supplementary video 8** The beating of cardiac spheroid on traditional hanging drop.

**Supplementary video 9** The beating of cardiac spheroid in low‐attachment plate.

**Supplementary video 10** Medium exchanging during spheroid culture.

**Supplementary video 11** The beating frequency changes before isoproterenol treatment (0 μm).

**Supplementary video 12** The beating frequency changes after isoproterenol treatment (0 μm).

**Supplementary video 13** The beating frequency changes before isoproterenol treatment (0.01 μm).

**Supplementary video 14** The beating frequency changes after isoproterenol treatment (0.01 μm).

**Supplementary video 15** The beating frequency changes before isoproterenol treatment (1 μm).

**Supplementary video 16** The beating frequency changes after isoproterenol treatment (1 μm).

## Data Availability

All data generated or analyzed during this study are included in this published article [and its supplementary information files].

## References

[1] Nguyen DC , Hookway TA , Wu Q , et al. Microscale generation of cardiospheres promotes robust enrichment of cardiomyocytes derived from human pluripotent stem cells. Stem Cell Reports. 2014;3:260‐268. doi:10.1016/j.stemcr.2014.06.002 25254340 PMC4175548

[2] Jiang B , Xiang Z , Ai Z , et al. Generation of cardiac spheres from primate pluripotent stem cells in a small molecule‐based 3D system. Biomaterials. 2015;65:103‐114. doi:10.1016/j.biomaterials.2015.06.024 26148474

[3] Zuppinger C . 3D culture for cardiac cells. Biochim Biophys Acta. 2016;1863:1873‐1881. doi:10.1016/j.bbamcr.2015.11.036 26658163

[4] Kokkinopoulos I et al. Cardiomyocyte differentiation from mouse embryonic stem cells using a simple and defined protocol. Dev Dyn. 2016;245:157‐165. doi:10.1002/dvdy.24366 26515123

[5] Mercola M , Ruiz‐Lozano P , Schneider MD . Cardiac muscle regeneration: lessons from development. Genes Dev. 2011;25:299‐309. doi:10.1101/gad.2018411 21325131 PMC3042154

[6] Beauchamp P et al. Development and characterization of a scaffold‐free 3D spheroid model of induced pluripotent stem cell‐derived human cardiomyocytes. Tissue Eng Part C Methods. 2015;21:852‐861. doi:10.1089/ten.TEC.2014.0376 25654582

[7] Zuppinger C . 3D cardiac cell culture: a critical review of current technologies and applications. Front Cardiovasc Med. 2019;6:87. doi:10.3389/fcvm.2019.00087 31294032 PMC6606697

[8] Vadivelu R , Kamble H , Shiddiky M , Nguyen N‐T . Microfluidic technology for the generation of cell spheroids and their applications. Micromachines. 2017;8:94. doi:10.3390/mi8040094

[9] Takeda M , Miyagawa S , Fukushima S , et al. Development of in vitro drug‐induced cardiotoxicity assay by using three‐dimensional cardiac tissues derived from human induced pluripotent stem cells. Tissue Eng Part C Methods. 2018;24:56‐67. doi:10.1089/ten.TEC.2017.0247 28967302 PMC5757089

[10] Richards DJ , Li Y , Kerr CM , et al. Human cardiac organoids for the modelling of myocardial infarction and drug cardiotoxicity. Nat Biomed Eng. 2020;4:446‐462. doi:10.1038/s41551-020-0539-4 32284552 PMC7422941

[11] Stevens KR , Pabon L , Muskheli V , Murry CE . Scaffold‐free human cardiac tissue patch created from embryonic stem cells. Tissue Eng Pt A. 2009;15:1211‐1222. doi:10.1089/ten.tea.2008.0151 PMC2774496 19063661

[12] Stevens KR , Kreutziger KL , Dupras SK , et al. Physiological function and transplantation of scaffold‐free and vascularized human cardiac muscle tissue. Proc Natl Acad Sci USA. 2009;106:16568‐16573. doi:10.1073/pnas.0908381106 19805339 PMC2746126

[13] Mannhardt I , Breckwoldt K , Letuffe‐Brenière D , et al. Human engineered heart tissue: analysis of contractile force. Stem Cell Reports. 2016;7:29‐42. doi:10.1016/j.stemcr.2016.04.011 27211213 PMC4944531

[14] Sebastiao MJ et al. Bioreactor‐based 3D human myocardial ischemia/reperfusion in vitro model: a novel tool to unveil key paracrine factors upon acute myocardial infarction. Transl Res. 2020;215:57‐74. doi:10.1016/j.trsl.2019.09.001 31541616

[15] Polonchuk L , Chabria M , Badi L , et al. Cardiac spheroids as promising in vitro models to study the human heart microenvironment. Sci Rep. 2017;7:7005. doi:10.1038/s41598-017-06385-8 28765558 PMC5539326

[16] Beauchamp P , Jackson CB , Ozhathil LC , et al. 3D co‐culture of hiPSC‐derived cardiomyocytes with cardiac fibroblasts improves tissue‐like features of cardiac spheroids. Front Mol Biosci. 2020;7:14. doi:10.3389/fmolb.2020.00014 32118040 PMC7033479

[17] Yang B , Lui C , Yeung E , et al. A Net mold‐based method of biomaterial‐free three‐dimensional cardiac tissue creation. Tissue Eng Part C Methods. 2019;25:243‐252. doi:10.1089/ten.TEC.2019.0003 30913987 PMC13215059

[18] Qiao L , Dho SH , Kim JY , Kim LK . SEPHS1 is dispensable for pluripotency maintenance but indispensable for cardiac differentiation in mouse embryonic stem cells. Biochem Biophys Res Commun. 2022;590:125‐131. doi:10.1016/j.bbrc.2021.12.091 34974300

[19] Ong CS , Fukunishi T , Nashed A , et al. Creation of cardiac tissue exhibiting mechanical integration of spheroids using 3D bioprinting. J Vis Exp. 2017;125. doi:10.3791/55438 PMC5608529 28715377

[20] Noguchi R , Nakayama K , Itoh M , et al. Development of a three‐dimensional pre‐vascularized scaffold‐free contractile cardiac patch for treating heart disease. J Heart Lung Transplant. 2016;35:137‐145. doi:10.1016/j.healun.2015.06.001 26433566

[21] Pointon A , Pilling J , Dorval T , Wang Y , Archer C , Pollard C . From the cover: high‐throughput imaging of cardiac microtissues for the assessment of cardiac contraction during drug discovery. Toxicol Sci. 2017;155:444‐457. doi:10.1093/toxsci/kfw227 28069985

[22] Devarasetty M , Forsythe S , Shupe T , et al. Optical tracking and digital quantification of beating behavior in bioengineered human cardiac organoids. Biosensors. 2017;7. doi:10.3390/bios7030024 PMC5618030 28644395

[23] Correia C , Koshkin A , Duarte P , et al. 3D aggregate culture improves metabolic maturation of human pluripotent stem cell derived cardiomyocytes. Biotechnol Bioeng. 2018;115:630‐644. doi:10.1002/bit.26504 29178315

[24] Archer CR , Sargeant R , Basak J , Pilling J , Barnes JR , Pointon A . Characterization and validation of a human 3d cardiac microtissue for the assessment of changes in cardiac pathology. Sci Rep. 2018;8:10160. doi:10.1038/s41598-018-28393-y 29976997 PMC6033897

[25] Kahn‐Krell A , Pretorius D , Guragain B , et al. A three‐dimensional culture system for generating cardiac spheroids composed of cardiomyocytes, endothelial cells, smooth‐muscle cells, and cardiac fibroblasts derived from human induced‐pluripotent stem cells. Front Bioeng Biotechnol. 2022;10:908848. doi:10.3389/fbioe.2022.908848 35957645 PMC9361017

[26] Kwon C , Arnold J , Hsiao EC , Taketo MM , Conklin BR , Srivastava D . Canonical Wnt signaling is a positive regulator of mammalian cardiac progenitors. Proc Natl Acad Sci USA. 2007;104:10894‐10899. doi:10.1073/pnas.0704044104 17576928 PMC1904134

[27] Richards DJ , Coyle RC , Tan Y , et al. Inspiration from heart development: Biomimetic development of functional human cardiac organoids. Biomaterials. 2017;142:112‐123. doi:10.1016/j.biomaterials.2017.07.021 28732246 PMC5562398

[28] Garzoni LR , Rossi MID , de Barros APDN , et al. Dissecting coronary angiogenesis: 3D co‐culture of cardiomyocytes with endothelial or mesenchymal cells. Exp Cell Res. 2009;315:3406‐3418. doi:10.1016/j.yexcr.2009.09.016 19769963

[29] Wu HW , Hsiao YH , Chen CC , Yet SF , Hsu CH . A PDMS‐based microfluidic hanging drop chip for embryoid body formation. Molecules. 2016;21:1‐11. doi:10.3390/molecules21070882 PMC6272923 27399655

[30] Rismani Yazdi S , Shadmani A , Bürgel SC , Misun PM , Hierlemann A , Frey O . Adding the'heart'to hanging drop networks for microphysiological multi‐tissue experiments. Lab Chip. 2015;15:4138‐4147. doi:10.1039/c5lc01000d 26401602 PMC5424877

[31] Rodoplu D , Matahum JS , Hsu CH . A microfluidic hanging drop‐based spheroid co‐culture platform for probing tumor angiogenesis. Lab Chip. 2022;22:1275‐1285. doi:10.1039/d1lc01177d 35191460

[32] Pettinato G , Wen X , Zhang N . Engineering strategies for the formation of embryoid bodies from human pluripotent stem cells. Stem Cells Dev. 2015;24:1595‐1609. doi:10.1089/scd.2014.0427 25900308 PMC4499791

[33] Yasuda E et al. Development of cystic embryoid bodies with visceral yolk‐sac‐like structures from mouse embryonic stem cells using low‐adherence 96‐well plate. J Biosci Bioeng. 2009;107:442‐446. doi:10.1016/j.jbiosc.2008.12.004 19332306

[34] Tanaka N , Takeuchi T , Neri QV , Sills ES , Palermo GD . Laser‐assisted blastocyst dissection and subsequent cultivation of embryonic stem cells in a serum/cell free culture system: applications and preliminary results in a murine model. J Transl Med. 2006;4:20. doi:10.1186/1479-5876-4-20 16681851 PMC1479373

[35] Sampurna B , Audira G , Juniardi S , Lai Y‐H , Hsiao C‐D . A simple imagej‐based method to measure cardiac rhythm in zebrafish embryos. Inventions. 2018;3:21. doi:10.3390/inventions3020021

