# Supplementary material for: The hanging‐heart chip: A portable microfluidic device for high‐throughput generation of contractile embryonic stem cell‐derived cardiac spheroids
Source: Bioeng Transl Med. 2024 Oct 8;10(3):e10726. doi: 10.1002/btm2.10726 (PMC12079401; doi:10.1002/btm2.10726)
Supplement: Supplementary file 1 — Supplementary Figure 1. Images of ES cells suspension in the microfluidic chip at (a) 3 × 10,4 (b) 6 × 10,4 and (c) 9 × 10^4^ cell/mL loading densities. Scale bar = 1000 μm. Supplementary Figure 2. Photographs of ES spheroids cultured in the microfluidic chip for 7 days at (a) 3 × 10,4 (b) 6 × 10,4 and (c) 9 × 10^4^ cell/mL loading densities, and for 15 days at (d) 3 × 10,4 (e) 6 × 10,4 and (f) 9 × 10^4^ cell/mL loading densities. Scale bar = 4000 μm. Supplementary Figure 3. (a) ACTN2 and (b) cTnT protein expression of cardiac spheroids cultured in the HH‐chip for 15 days. Data are presented as the mean ± SD, n >19, in three independent experiments. Supplementary Figure 4. The percentage of cardiomyocytes in individual spheroids was quantified by the intensity of (a) ACTN2 and (b) cTnT divided by the intensity of total cells stained with Hoechst at different cell seeding densities. Data are presented as the mean ± SD, n = 6, in three independent experiments. Supplementary Figure 5. Images of ES cells cultured by using HH‐Chip (left column), traditional hanging drop (middle column), and low‐attachment 96‐well plate (right column) methods. Scale bar = 1000 μm. Supplementary Figure 6. Photographs of ES spheroids formed in the commercial low‐attachment 96‐well plate after 7 days of culture. Scale bar = 10 mm. Supplementary Figure 7. Micrographs of mouse ES‐formed spheroids in traditional hanging drops for 7 days. The arrows indicate the spheroids that attached to the supporting surface due to manual pipetting during medium change. Scale bar = 1000 μm. Supplementary Figure 8. The percentage of cardiomyocytes in individual spheroids was quantified by the intensity of (a) ACTN2 and (b) cTnT divided by the intensity of total cells stained with Hoechst in three spheroid culture methods. Data are presented as the mean ± SD, n = 6, in three independent experiments. Supplementary Figure 9. (a) The beating frequency of a cardiac spheroid measured at the selecting region which is shown [file BTM2-10-e10726-s013.docx]

­

**Supplementary Figure 1.** Images of ES cells suspension in the microfluidic chip at (a) 3 × 10^4^, (b) 6 × 10^4^, and (c) 9 × 10^4^ cell/mL loading densities. Scale bar = 1000 μm.

**Supplementary Figure 2.** Photographs of ES spheroids cultured in the microfluidic chip for 7 days at (a) 3 × 10^4^, (b) 6 × 10^4^, and (c) 9 × 10^4^ cell/mL loading densities, and for 15 days at (d) 3 × 10^4^, (e) 6 × 10^4^, and (f) 9 × 10^4^ cell/mL loading densities. Scale bar = 4000 μm.

**Supplementary Figure 3.** (a) ACTN2 and (b) cTnT protein expression of cardiac spheroids cultured in the HH-chip for 15 days. Data are presented as the mean ± SD, n > 19, in three independent experiments.

**Supplementary Figure 4.** The percentage of cardiomyocytes in individual spheroids was quantified by the intensity of (a) ACTN2 and (b) cTnT divided by the intensity of total cells stained with Hoechst at different cell seeding densities. Data are presented as the mean ± SD, n = 6, in three independent experiments.

**Supplementary Figure 5.** Images of ES cells cultured by using HH-Chip (left column), traditional hanging drop (middle column), and low-attachment 96-well plate (right column) methods. Scale bar = 1000 μm.


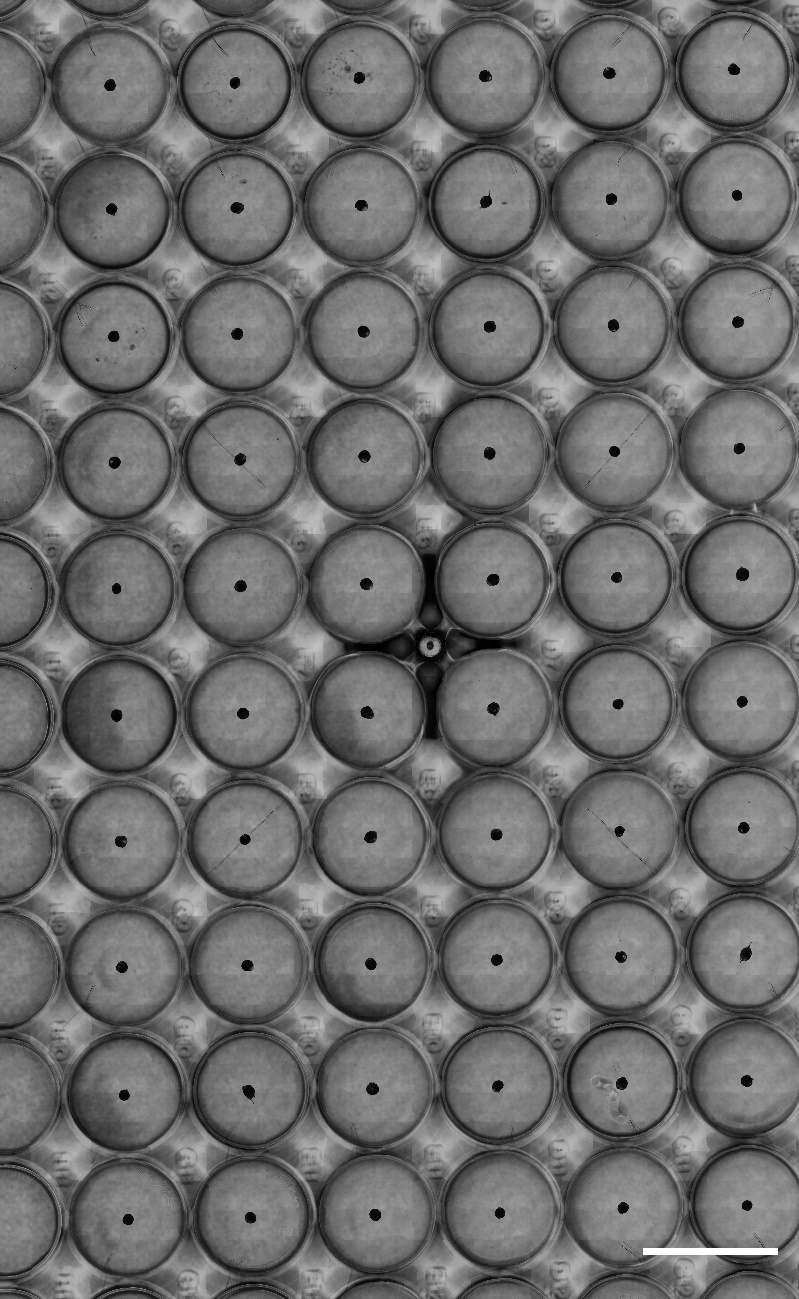


**Supplementary Figure 6.** Photographs of ES spheroids formed in the commercial low-attachment 96-well plate after 7 days of culture. Scale bar = 10 mm.

**Supplementary Figure 7.** Micrographs of mouse ES-formed spheroids in traditional hanging drops for 7 days. The arrows indicate the spheroids which attached to the supporting surface due to manual pipetting during medium change. Scale bar = 1000 μm.

**Supplementary Figure 8.** The percentage of cardiomyocytes in individual spheroids was quantified by the intensity of (a) ACTN2 and (b) cTnT divided by the intensity of total cells stained with Hoechst in three spheroid culture methods. Data are presented as the mean ± SD, n = 6, in three independent experiments

**Supplementary Figure 9.** (a) The beating frequency of a cardiac spheroid measured at the selecting region which is shown as the dashed circle in (b).
